# Investigating the water availability hypothesis of pot binding: small pots and infrequent irrigation confound the effects of drought stress in potato (*Solanum tuberosum* L.)

**DOI:** 10.3389/fpls.2024.1399250

**Published:** 2024-06-13

**Authors:** Dominic Hill, Lorenzo Conte, David Nelson, John Hammond, Luke Bell

**Affiliations:** ^1^ School of Agriculture, Policy and Development, University of Reading, Reading, United Kingdom; ^2^ Branston Ltd., Lincoln, United Kingdom

**Keywords:** pot binding, water availability hypothesis, drought stress, high-throughput phenotyping, multispectral imaging, experimental validity, potato, *Solanum tuberosum*

## Abstract

To maximise the throughput of novel, high-throughput phenotyping platforms, many researchers have utilised smaller pot sizes to increase the number of biological replicates that can be grown in spatially limited controlled environments. This may confound plant development through a process known as “pot binding”, particularly in larger species including potato (*Solanum tuberosum*), and under water-restricted conditions. We aimed to investigate the water availability hypothesis of pot binding, which predicts that small pots have insufficient water holding capacities to prevent drought stress between irrigation periods, in potato. Two cultivars of potato were grown in small (5 L) and large (20 L) pots, were kept under polytunnel conditions, and were subjected to three irrigation frequencies: every other day, daily, and twice daily. Plants were phenotyped with two Phenospex PlantEye F500s and canopy and tuber fresh mass and dry matter were measured. Increasing irrigation frequency from every other day to daily was associated with a significant increase in fresh tuber yield, but only in large pots. This suggests a similar level of drought stress occurred between these treatments in the small pots, supporting the water availability hypothesis of pot binding. Further increasing irrigation frequency to twice daily was still not sufficient to increase yields in small pots but it caused an insignificant increase in yield in the larger pots, suggesting some pot binding may be occurring in large pots under daily irrigation. Canopy temperatures were significantly higher under each irrigation frequency in the small pots compared to large pots, which strongly supports the water availability hypothesis as higher canopy temperatures are a reliable indicator of drought stress in potato. Digital phenotyping was found to be less accurate for larger plants, probably due to a higher degree of self-shading. The research demonstrates the need to define the optimum pot size and irrigation protocols required to completely prevent pot binding and ensure drought treatments are not inadvertently applied to control plants.

## 1 Introduction

In the last two decades, the rapid development of plant phenotyping technologies has alleviated a significant bottleneck in our understanding of, and ability to select for, desirable traits in important agricultural crops. Historically, the measurement of even simple phenotypic traits was often destructive, expensive, and time-consuming (Furbank and Tester, 2011). Now, non-destructive plant phenotyping can occur over agriculturally relevant areas and timescales, with comparatively low financial and labour costs (Pieruschka and Schurr, 2019).

Researchers have begun to combine these high-throughput phenotyping platforms (HTPPs) with controlled environments to understand how predicted climate scenarios might affect crops in the future (Langstroff et al., 2022). However, while the phenotyping bottleneck has been released, controlled environments with the requisite precision to maintain forecast conditions remain spatially limited. This has led to a trade-off between biological replication and the representativeness of laboratory-grown plants to their field-grown relatives.

To maximise replication in controlled environments and other confined spaces, many researchers utilise smaller pot sizes. However, small pots may confound plant development through a poorly understood process called “pot binding”. The “water availability” hypothesis of pot binding suggests that all plants in small pots are inadvertently drought stressed, as the small volumes of substrate hold insufficient amounts of freely extractible water to prevent this stress between irrigation periods (Sinclair et al., 2017).

If this process occurs in an experiment aiming to investigate the effects of water deficits on plant development, then pot binding will covertly increase the drought stress severity of both the water deficit treatment and the supposedly well-watered control treatment. As the severity of survivable drought stress is limited by the minimum volume of water available for transpiration (Turner, 2019), pot binding will therefore decrease the difference in water deficit between treatments.

This is particularly problematic for large crops with high water requirements, including potato (*Solanum tuberosum*). While potato has a high water-efficiency (Sun et al., 2015), it requires high volumes of water for efficient growth (Knox et al., 1997; Byrd et al., 2014; Knox et al., 2018) and is extremely susceptible to drought stress (Schafleitner et al., 2009). According to the water availability hypothesis, this increases the susceptibility of potato to pot binding, relative to the experimental pot size.

Previous research has aimed to provide guidelines to both impose meaningful drought stress (Turner, 2019) and prevent pot binding (Poorter et al., 2012) in pot experiments. The established recommendation to prevent pot binding, based on a meta-analysis of 65 studies, is that the ratio of dry plant biomass to substrate volume should not exceed 1 g L-1 (Poorter et al., 2012). As potato has been recorded to produce over 1,000 g of dry matter in controlled environments (Wheeler and Tibbitts, 1987), the recommendation would require a minimum pot volume of 1000 L, which is impractical for phenotyping experiments.

Previous research has aimed to create a more realistic recommendation for pot experiments with potato (Hill et al., 2023)^1^. Five pot sizes (2.5, 5, 10, 20, and 40 L) were used to investigate the confounding effects of pot size on water-restriction in potato and the practicalities of using larger pot sizes for phenotyping experiments. It was found that pots ≤ 5 L were inappropriate for investigating the effects of water-restriction on potato, primarily due to a strong drought-independent stunting effect observed in one of two cultivars evaluated, which was not seen in larger pot sizes. Large 40 L pots were also found to be impractical for controlled-environment studies where pots must be manually moved for phenotyping.

Here we investigate the water availability hypothesis in potato and assess whether the effects of pot binding on potato morphophysiology could be mitigated in practical pot sizes by reducing the inter-irrigation period. We also validate the specific phenotyping methods used by comparing their results with low-tech, established (Elsayed et al., 2021; Ninanya et al., 2021; Mthembu et al., 2022), and accurate methods.

## 2 Materials and methods

### 2.1 Plant material and growing conditions

A pot experiment was carried out at the Crop and Environment Laboratory (51°26’13”N 0°56’32.5”W) at the University of Reading, UK. Thirty pots of each size, 5 and 20 L, were filled with a 2:1 by volume mixture of John Innes No. 2 compost and sharp sand (Jubilee Building Supplies, Bracknell, UK). On 1^st^ June 2023, pre-sprouted seed tubers of both *Solanum tuberosum* cvs. Maris Piper and Charlotte were planted in individual pots, with 15 tubers planted into 5 L pots and 15 tubers planted into 20 L pots for each cultivar. All plants were grown outside and uncovered from planting until 28 days after planting (DAP) when they were moved under an open-ended polytunnel. Before being covered, all plants were grown under rainfed conditions with supplementary hand-watering to saturation when rainfall was insufficient. Once covered, all plants were irrigated to saturation daily with a manual irrigation system, until the start of the treatment conditions.

On 3^rd^ July (32 DAP), plants from each pot size and cultivar were randomly assigned to one of three water treatments: irrigation to saturation every other day (T_1/2_), irrigation to saturation daily (T_1_), or irrigation to saturation twice daily (T_2_). Each treatment comprised of 5 pots per pot size and cultivar. The maximum water lost from each pot size had previously been measured gravimetrically at 6-, 18-, 24-, and 48-hours post-saturation, at the cessation of excess runoff from each pot. From 3^rd^ July to 4^th^ August (64 DAP), all plants were automatically irrigated with the irrigation volumes in **Table 1**. After 4^th^ August, irrigation was withdrawn, the plant canopies were harvested, and the tubers were left to mature *in situ* until 18^th^ August (78 DAP).

**Table 1:** The three water treatments in this experiment were imposed with an automatic irrigation system that provided water to the pots at either 48-, 24-, or 18- and 6-hour intervals.

Treatment	5 L pots	20 L pots
Every Other Day (T_1/2_)	400 ml at 12:00	2,800 ml at 12:00
Daily (T_1_)	400 ml at 12:00	2,800 ml at 12:00
Twice Daily (T_2_)	400 ml at 12:00 + 200 ml at 18:00	2,800 ml at 12:00 + 800 ml at 18:00

Irrigation volumes were calculated gravimetrically as the maximum water lost from each pot size over the relevant time intervals. These conditions were imposed from 3^rd^ July to 4^th^ August, after a period of uniform well-watered conditions from planting.

### 2.2 Non-destructive data collection

Between 27^th^ June and 4^th^ August, average canopy temperature and SPAD values were recorded for each plant at least three times per week. Canopy temperatures were measured with an AIR-801 infrared thermometer with a resolution of 0.1°C (ATP Instrumentation, Ashby-de-la-Zouch, UK) and SPAD values were measured with SPAD-502Plus (Konica-Minolta, Tokyo, Japan). Three leaves, each from distinct levels within the canopy, were sampled and averaged to give an accurate estimate for the whole canopy (Víg et al., 2012). Canopy levels were standardised by measuring the terminal leaflet on the third highest fully expanded leaf (Gervais et al., 2021), followed by the terminal leaflets on the fifth and seventh leaves. To control for order effects, particularly on canopy temperature, measurements were taken from the highest canopy level of each plant first, followed by the second level, followed by the third. All measurements were taken between 10:00 and 12:00, to prevent the onset of irrigation from confounding the results.

On 13^th^ July (42 DAP), subsamples of three plants per group (treatment x pot size x cultivar) were scanned with two PlantEye F500 multispectral 3D scanners (Phenospex, Heerlen, Netherlands). It was anticipated that plants in the 20 L pots would grow too large to be accurately phenotyped by the PlantEye sensor. Therefore, subsamples of three plants per group were scanned for both pot sizes to maximise coverage for each plant and to maintain balance across the groups. PlantEye scanners have previously been used to measure “high-temperature-induced” (Lazarević et al., 2022) and drought-related (Hill et al., 2023)^1^ morphophysiological changes in potato. The PlantEye measured reflectance of five wavelengths: red (620–645 nm), green (530–540 nm), blue (460–485 nm), near-infrared (820–850 nm), and infrared (940 nm). The integrated software, Phena (Phenospex, Heerlen, Netherlands), generated 3D point clouds of the plants by triangulating adjacent points. These point clouds were then used by the HortControl software (Phenospex, Heerlen, Netherlands) to calculate morphological parameters, including digital biomass, greenness index (greenness), hue, normalised difference vegetation index (NDVI), plant senescence reflectance index (PSRI), leaf angle, and light penetration depth. Vegetation indices were calculated in HortControl as ratios of the reflectance of relevant wavelengths, e.g., greenness was calculated as $\frac{\left(2\;\times \;R_{Green}\;–\;R_{Red}\;–\;R_{Blue}\right)}{\left(R_{Green}\;+\;R_{Red}\;+\;R_{Blue}\right)}$ , where *R* equals reflectance. Morphological parameters were calculated from the spatial distribution of triangles within the point clouds (Lazarević et al., 2021). Due to the high correlations between certain variables, e.g., digital biomass, leaf area, and leaf area index, only the previously stated variables were analysed.

### 2.3 Destructive data collection

All plant canopies were harvested on 4^th^ August 2023, 64 DAP. Fresh canopy biomass was measured immediately post-harvest. Canopies were then individually bagged and oven-dried at 60°C for at least 72 hours. The canopies were then reweighed for the calculation of canopy dry matter percentage. Tubers were left to mature *in situ* for an additional 14 days, after which they were counted and weighed. Subsamples of three representative tubers per plant were sliced into 5 mm cross-sections, and oven-dried at 60°C for at least 72 hours. The sliced tubers were then reweighed to calculate tuber dry matter content.

### 2.4 Statistical analysis

All statistical analyses were completed in RStudio (RStudio Team, 2020). For each relevant dependent variable, a linear model was generated with either the formula treatment x pot size x cultivar or treatment x pot size x cultivar x sample date, depending on whether that variable was measured once or over time. QQ plots and Shapiro-Wilks test of normality were used on the residuals of each model to check that the assumption of normality was met. Shapiro-Wilks tests were also used to check the assumption of normality by groups. The assumption of homogeneity of variance was checked using Levene’s test with the same formula as each respective model. Average canopy temperature and average canopy SPAD were assessed for normality with histograms and QQ plots exclusively as the sample sizes for these variables were too high to be accurately assessed for normality with Shapiro-Wilks tests (Lumley et al., 2002). If any of these assumptions were not met, signified by a *p*-value ≤ 0.05 or a non-normal QQ plot, the data were transformed, and the tests of normality and homogeneity of variance were reassessed.

Once these assumptions were met, a three or four-way ANOVA was run on the model for each variable. Average canopy temperature and canopy SPAD were assessed with repeated measures ANOVAs, with sample date as a within-subjects factor and plant numbers as unique identifiers. The data for these assessments were found to violate the assumption of sphericity, due to the high number of repeat measurements, so an appropriate correction was applied (Haverkamp and Beauducel, 2017). The Greenhouse-Geisser (GG) correction was selected as it has recently been demonstrated to be more conservative than Huynh-Feldt adjustments (Blanca et al., 2023). Any significant interactions, signified by a *p*-value ≤ 0.05, were decomposed into simple three and/or two-way interactions and simple main effects, all with appropriate Bonferroni adjustments. When the assumptions of the ANOVA were met, the overall error term from each ANOVA was used for all further analysis of that dependent variable.

All data presented here refer to estimated marginal means that were extracted from the linear model for the respective dependent variable with the “emmeans” package in R. These means, ± 95% CIs were then used to represent the data graphically with the “ggplot2” package. Any data that required transformation to meet the assumptions of the relevant statistical tests were back transformed with the inverse function before being represented graphically. Compact letters were calculated from the estimated marginal means and 95% CIs with the “multcomp” package. Means not sharing any letter are significantly different by the Tukey-test at the 0.05% level of significance (Piepho, 2018). To ensure the consistency of language and comparisons, differences between means are presented here as absolute values and percentage differences, i.e., the difference between the two means divided by their average.

To compare the two methods of measuring biomass, digitally and gravimetrically, the data were split into two groups based on pot size. The data were then filtered to exclude the two plants from each group that were not scanned on 13^th^ July. Both measurements were then assessed for normality with Shapiro-Wilks tests and QQ plots. Once normality was assured, correlation coefficients and *p*-values for each pot size were calculated with the Pearson method.

## 3 Results

### 3.1 Manual tuber measurements

#### 3.1.1 Pot size significantly affects fresh tuber yield, but irrigation treatment only has a significant effect in larger pots

Mean fresh tuber yield (FTY) was significantly affected by pot size (*p*< 0.001) and cultivar (*p*< 0.001), but not by treatment (*p* = 0.081) (**Table 2**, **Supplementary Table S1**). There was also a single significant interaction effect between pot size and treatment (*p* = 0.003) on FTY. Pot size had the greatest effect of the three grouping factors; there was a large (836.8 g, 117.1%) difference in FTY across all plants in 20 L pots $\left(\bar{x}=1132.8\;g\right)$ compared to 5 L pots $\left(\bar{x}=296.0\;g\right)$ . The difference between cultivars was much smaller (71.8 g, 13%); the mean FTY of all Maris Piper plants $\left(\bar{x}=683.1\;g\right)$ was slightly higher than that of Charlotte $\left(\bar{x}=611.3\;g\right)$ .

**Table 2:** Main effects and interaction terms of a three-way ANOVA for fresh tuber yield (√(g)), mean tuber mass (log10(g)), tuber dry matter (%), fresh canopy biomass (log10(g)), canopy dry matter (log10(%)) of two potato cultivars (Maris Piper and Charlotte), grown in one of two pots sizes (5 and 20 L) and subjected to every other day, daily, or twice daily irrigation treatments.

Effect	DF	Fresh Tuber Yield (√(g))		Mean Tuber Mass (log10(g))		Tuber Dry Matter (%)		Fresh Canopy Biomass (log10(g))		Canopy Dry Matter (log10(%))	
		F	p	F	p	F	p	F	p	F	p
Treatment (T)	47	2.7	0.081	0.7	0.486	0.7	0.499	27.2	**0.000**	286.4	**0.000**
Pot Size (PS)	47	2069.6	**0.000**	39.4	**0.000**	14.5	**0.000**	3621.2	**0.000**	17.8	**0.000**
Cultivar (C)	47	19.5	**0.000**	0.1	0.733	100.8	**0.000**	40.4	**0.000**	6.7	**0.003**
T x PS	47	6.8	**0.003**	0.4	0.660	1.1	0.334	3.4	**0.043**	32.5	**0.000**
T x C	47	1.0	0.372	0.1	0.926	1.1	0.329	0.2	0.840	1.3	0.270
PS x C	47	2.7	0.106	3.1	0.082	9.7	**0.003**	3.2	0.078	3.2	0.051
T x PS x C	47	2.2	0.127	0.4	0.694	2.1	0.132	0.2	0.828	0.5	0.620

Significant p-values (< 0.05) are indicated in bold.

**Figure 1:**
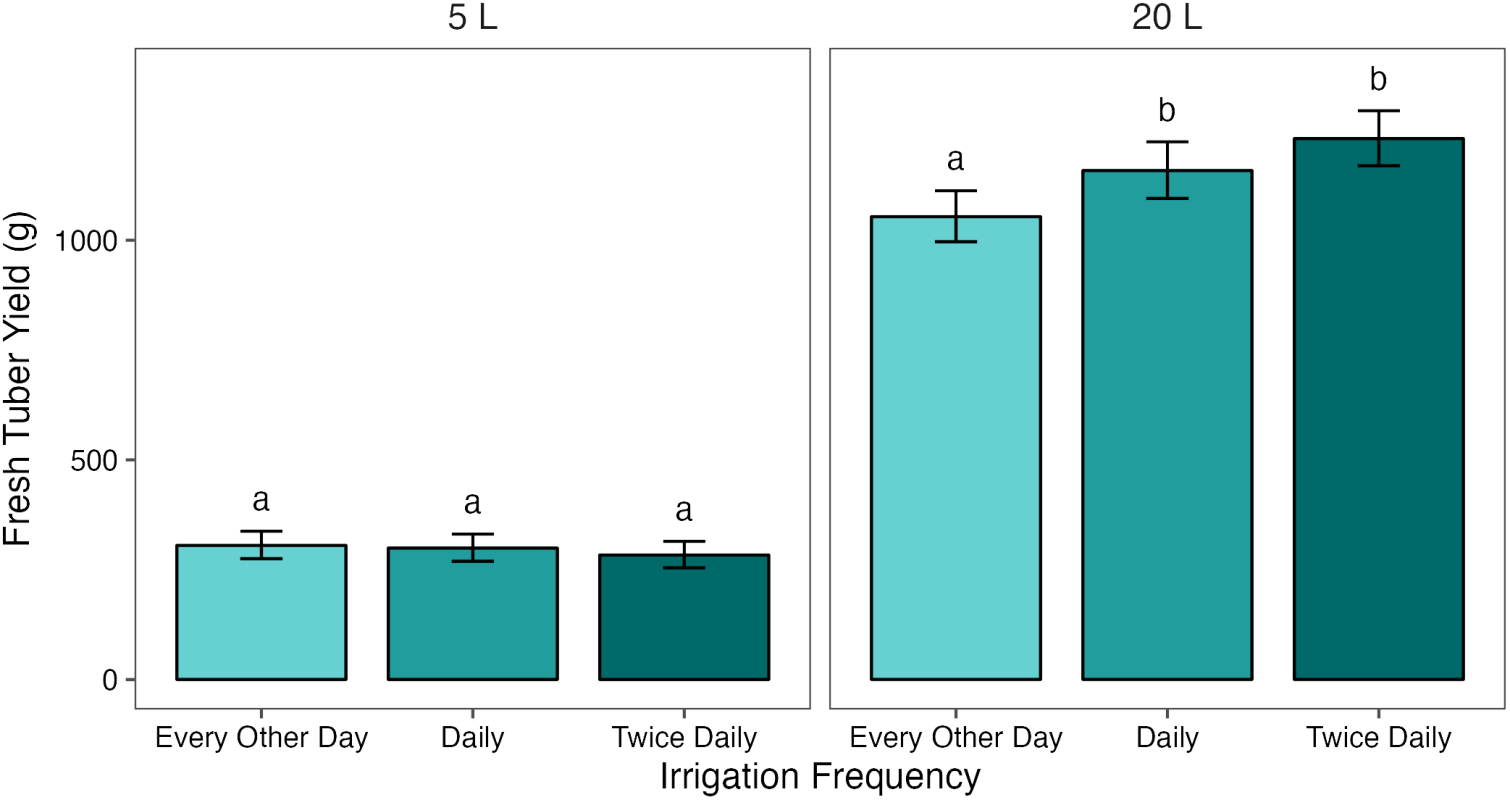
Mean fresh tuber yields (FTY) from potato plants grown in two pot sizes, 5 and 20 L, under three different water treatments: watered to capacity every other day, daily, and twice daily. Plants were grown under an open-ended polytunnel between 1st June and 4th August 2023. Tubers were harvested on 18th August 2023, 78 days after planting. Means represent FTY across two cultivars of potato, Maris Piper and Charlotte, (n = 10) ± 95% CIs. Means with different letters within each panel were significantly different by Tukey’s test (p< 0.05).

When grouped by pot size, treatment had a significant effect on FTY in 20 L pots (*p* = 0.001), but not in 5 L pots (*p* = 1.000). The response to treatment in 20 L pots was dose-dependent (**Figure 1**). Each increase in irrigation frequency was associated with an increase in FTY, but only the difference between T_1/2_ and T_1_ or T_2_ was significant (*p*< 0.05). There was a small (63.0 g, 5.8%) difference in FTY between 20 L pots under T_1/2_ $\left(\bar{x}=1053.7\;g\right)$ and T_1_ $\left(\bar{x}=1116.8\;g\right)$ , and a larger (114.7 g, 9.8%) difference between the latter and pots under T_2_ $\left(\bar{x}=1231.4\;g\right)$ .

The analysis of tuber number demonstrated a lack of within-group normality due to the incongruently consistent tuber number within a single group (T_1/2_, 5 L, Charlotte). In this group, all but one replicate produced 10 tubers, the other produced 7, causing a significantly non-normal distribution (*p*< 0.001) that was not present across the whole sample or within any other groups. Further statistical analysis was discarded, but simple summary statistics showed only pot size had a noticeable effect on tuber number; plants in 5 L pots produced an average of 10 tubers, compared to 25 tubers in 20 L pots. When grouped by either cultivar or treatment, average tuber number per plant was within one tuber for each group.

There was sufficient variation in FTY within the non-normal group that further analysis of mean tuber mass was appropriate. Mean tuber mass was only significantly affected by pot size (*p*< 0.001), with no statistically significant interactions (**Table 2**). There was a small (13.4 g, 35.8%) difference between the pot sizes; the mean tuber mass of all plants in 20 L pots $\left(\bar{x}=44.0\;g\right)$ was slightly higher than that in 5 L pots $\left(\bar{x}=30.6\;g\right)$ .

In summary, mean fresh tuber yield (FTY) was significantly affected by pot size and cultivar, but not by treatment (**Table 2**, **Supplementary Table S1**). Pot size had the greatest effect on FTY, with an 836.8 g (117.1%) difference between FTY in 5 and 20 L pots; FTY was greater in the latter. Treatment had a significant effect exclusively in 20 L pots, with each increase in irrigation frequency being associated with an increase in FTY, although the difference in FTY between plants irrigated daily and twice daily was not significant in this pot size (**Figure 1**). Mean tuber mass was also significantly affected by pot size, but the effect was small (13.4 g, 35.8%).

#### 3.1.2 Pot size significantly affects tuber dry matter in Maris Piper, but not in Charlotte

Mean tuber dry matter percentage (TDM%) was significantly affected by pot size (*p*< 0.001) and cultivar (*p*< 0.001), but not by treatment (*p* = 0.499). There was also a significant interaction between pot size and cultivar (*p* = 0.003) (**Table 2**). Cultivar had the greatest effect of the three grouping factors; there was a small (3%, 14.3%) difference in TDM% between Maris Piper $\left(\bar{x}=20.7\%\right)$ and Charlotte $\left(\bar{x}=18.0\%\right)$ . The difference between pot sizes was smaller (1.2% 6.5%); the mean TDM% of all plants in 5 L pots $\left(\bar{x}=20\%\right)$ was very slightly higher than of those in 20 L pots $\left(\bar{x}=19\%\right)$ .

The interaction effect between pot size and cultivar demonstrated a difference in the effect of pot size on TDM% between the two cultivars. There was no significant (*p* = 1.000) difference in the TDM% of Charlotte between the 5 L $\left(\bar{x}=18.0\%\right)$ and 20 L pots $\left(\bar{x}=17.9\%\right)$ , but there was a significant (*p*< 0.001) difference (2.3%, 10.9) between Maris Piper in 5 L $\left(\bar{x}=21.9\%\right)$ and 20 L pots $\left(\bar{x}=19.6\%\right)$ . TDM% in Maris Piper was also significantly higher than that of Charlotte in both 5 L (*p*< 0.001) and 20 L (*p*< 0.001) pots.

### 3.2 Manual canopy measurements

#### 3.2.1 Increasing irrigation from every other day to daily significantly increases canopy biomass, but further increases have no significant effect

Fresh canopy biomass was significantly affected by pot size (*p*< 0.001), cultivar (*p*< 0.001), and treatment (*p*< 0.001) (**Table 2**). There was also a significant interaction between pot size and treatment (*p* = 0.043). Pot size had the greatest effect on canopy biomass, with a very large (934.1 g, 191.9%) difference between 5 L $\left(\bar{x}=173.3\;g\right)$ and 20 L pots $\left(\bar{x}=953.7\;g\right)$ . Maris Piper $\left(\bar{x}=444.8\;g\right)$ produced heavier (73.3 g, 18.0%) canopies than Charlotte $\left(\bar{x}=371.5\;g\right)$ and each increase in irrigation frequency was associated with an average increase in fresh biomass, although these were not always significant.

**Figure 2:**
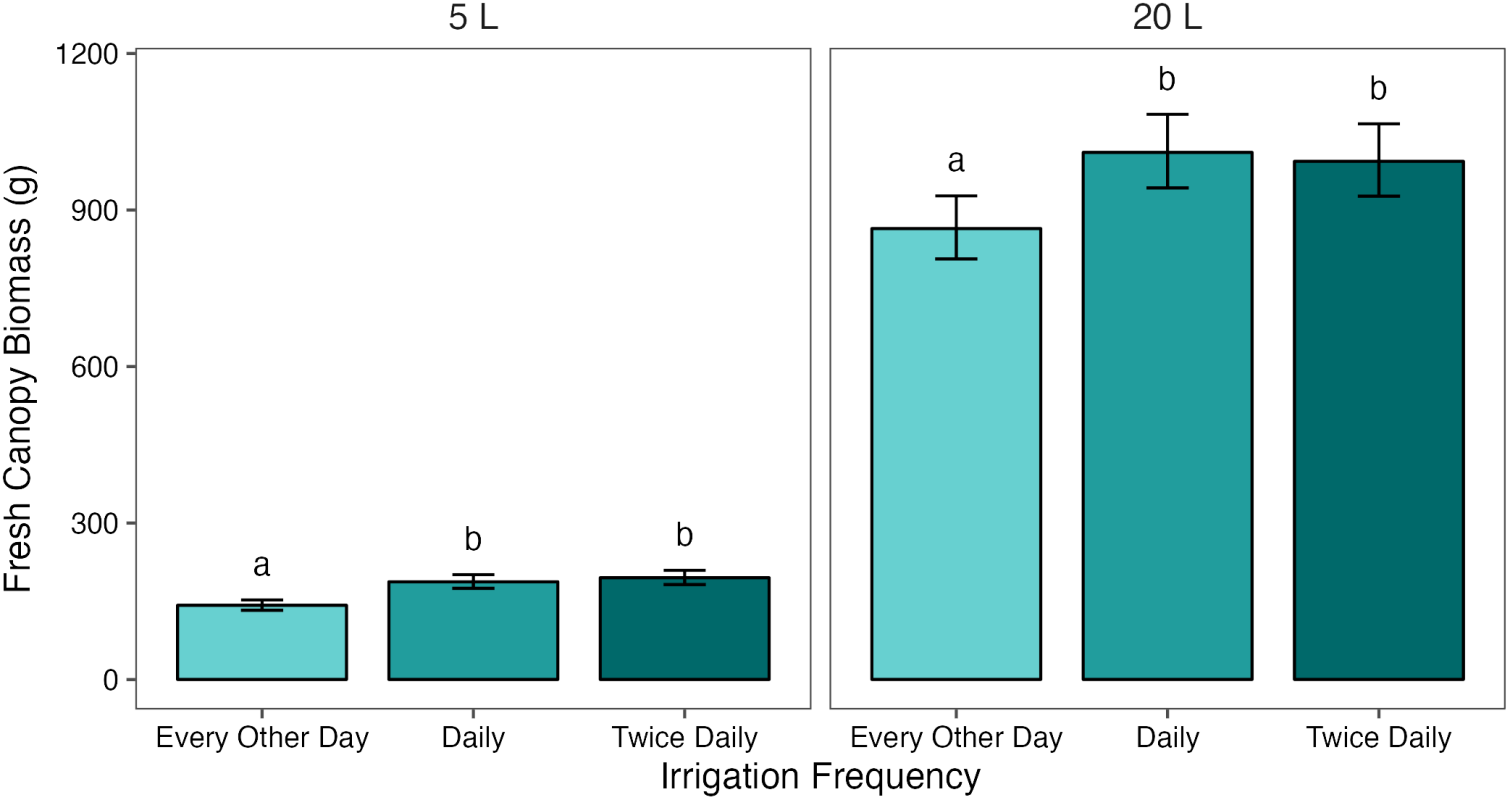
Mean fresh canopy biomass of potato plants grown in two pot sizes, 5 and 20 L, each under three different water treatments: watered to capacity every other day, daily, and twice daily. Plants were grown under an open-ended polytunnel between 1^st^ June and 4^th^ August 2023. Canopies were harvested on 4^th^ August, 64 days after planting. Means represent canopy biomass across two cultivars of potato, Maris Piper and Charlotte, (n = 10) ± 95% CIs. Means with different letters within each facet were significantly different by Tukey’s test (*p*< 0.05).

Analysis of the interaction effect between pot size and treatment showed that, when averaged across the two cultivars, canopy biomass increased significantly (*p*< 0.05) between T_1/2_ and T_1_ in both 5 L and 20 L pots, with a difference of 45.1 g (27.3%) and 129.0 g (13.9%) between treatments, respectively. In the 5 L pots, there was no significant difference in biomass between the T_1_ $\left(\bar{x}=187.4\;g\right)$ and T_2_ $\left(\bar{x}=195.1\;g\right)$ treatments. This effect was consistent in the 20 L pots, where there was no significant difference between the T_1_ $\left(\bar{x}=1010.3\;g\right)$ and T_2_ $\left(\bar{x}=993.4\;g\right)$ treatments (**Figure 2**).

#### 3.2.2 Pot size significantly affects canopy dry matter in Charlotte, but not in Maris Piper

Canopy dry matter percentage (CDM%) was significantly affected by all three grouping factors (pot size, *p*< 0.001; cultivar, *p*< 0.001; treatment, *p* = 0.003) and there was a significant interaction effect between pot size and cultivar (*p*< 0.001) (**Table 2**). When averaged across all treatments, pot size had a significant effect on the CDM% of Charlotte (*p*< 0.001), but not on that of Maris Piper (*p* = 0.590). The CDM% of Maris Piper in 5 L $\left(\bar{x}=10.5\%\right)$ and 20 L pots $\left(\bar{x}=10.8\%\right)$ were within 1%, while that of Charlotte was significantly lower in 20 L pots $\left(\bar{x}=6.7\%\right)$ than in 5 L pots $\left(\bar{x}=8.3\%\right)$ . Cultivar had the greatest effect on CDM%, with an absolute difference of 3.1% (34.7% difference), compared to a 0.8% (8.7% difference) between the pot sizes. Treatment had a smaller effect, with an absolute difference of 0.5% (5.7% difference) between plants under T_1/2_ and T_1_, and 0.3% (3.6% difference) between the latter and T_2_. Again, there was a significant difference in canopy dry matter percentage between T_1/2_ irrigation and T_2_ (*p*< 0.05), but not between either of those frequencies and T_1_ (*p* > 0.05).

#### 3.2.3 Each increase in irrigation frequency was associated with a significant decrease in canopy temperature

**Figure 3:**
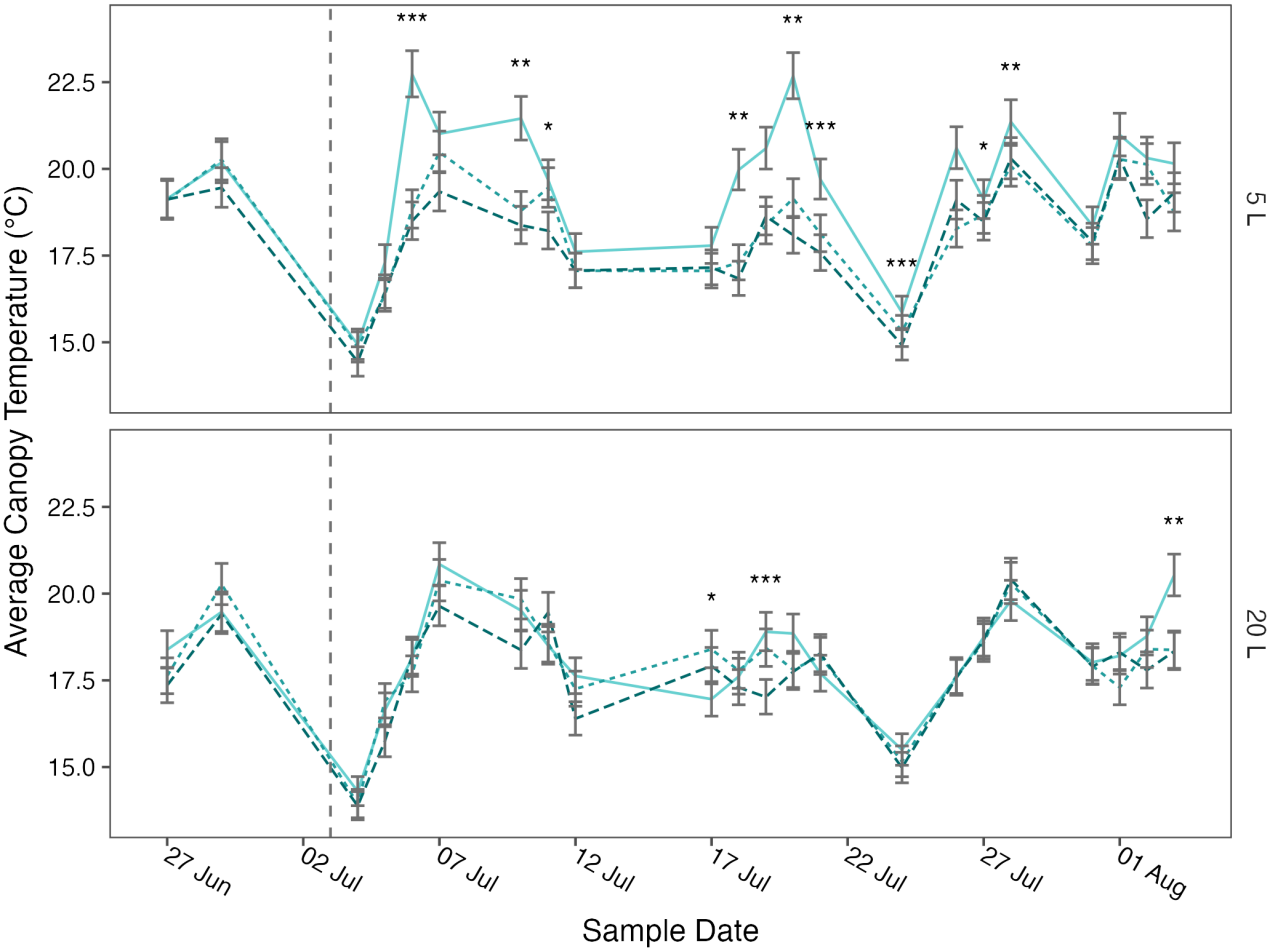
Mean canopy temperature of potato (cv. Maris Piper) over time, grown in two pot sizes, 5 (top) and 20 L (bottom), each under three water treatments: watered to capacity every other day (solid line), daily (dotted line), and twice daily (dashed line). Plants were grown under an open-ended polytunnel between 1^st^ June and 4^th^ August 2023. Canopy temperature was measured between 27^th^ June and 4^th^ August. The different irrigation frequency treatments commenced on 3^rd^ July 2023 (vertical dashed line). Means represent canopy temperature averaged across three canopy levels: top, middle, and bottom, (n = 5) ± 95% CIs. Means with asterisks above were significantly affected by treatment according to main effects analysis grouped by pot size, cultivar, and sample date with a Bonferroni p-value adjustment (* = *p*< 0.05, ** = *p*< 0.01, *** = *p*< 0.001).

**Figure 4:**
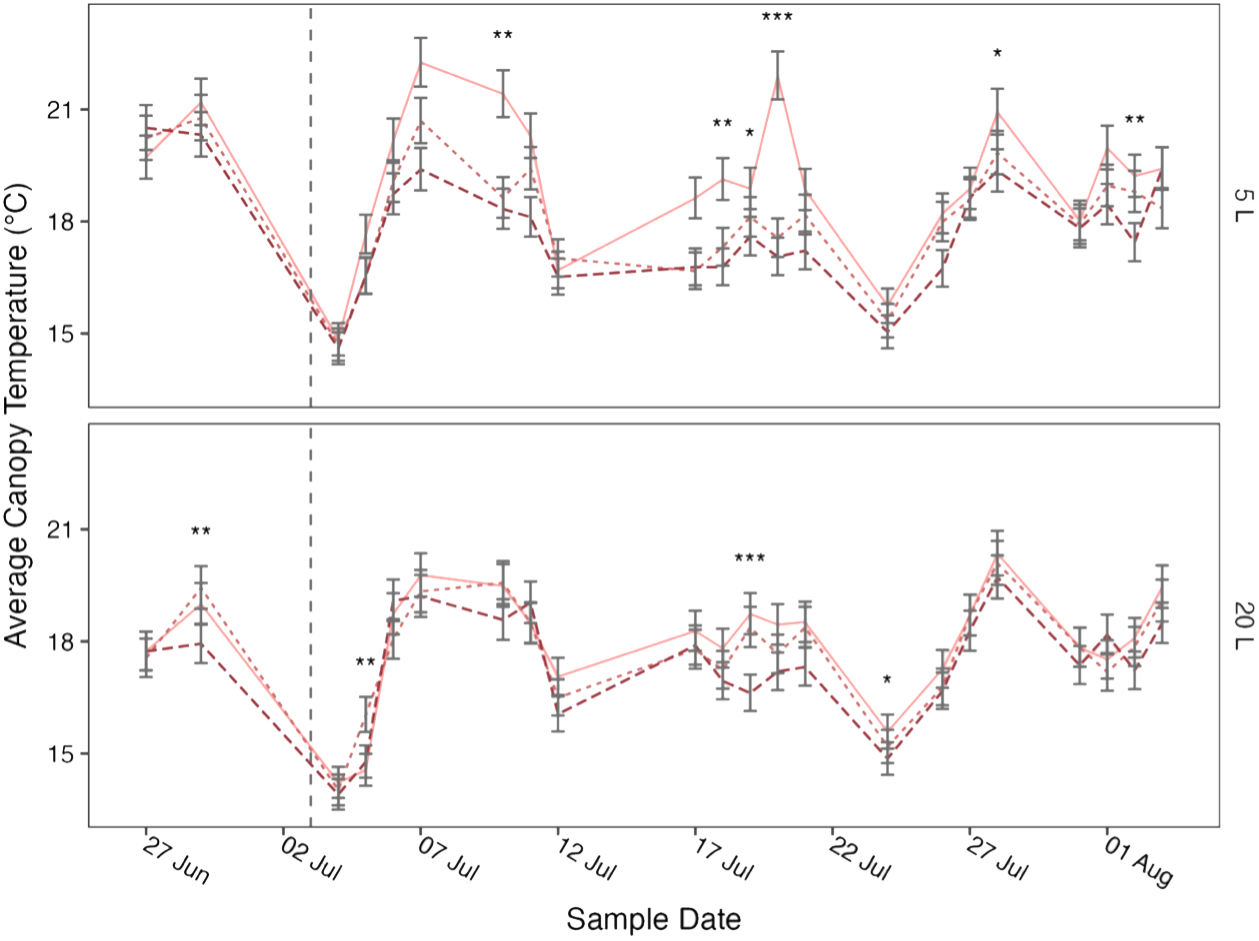
Mean canopy temperature of potato (cv. Charlotte) over time, grown in two pot sizes, 5 (top) and 20 L (bottom), each under three water treatments: watered to capacity every other day (solid line), daily (dotted line), and twice daily (dashed line). Plants were grown under an open-ended polytunnel between 1^st^ June and 4^th^ August 2023. Canopy temperature was measured between 27^th^ June and 4^th^ August. The different irrigation frequency treatments commenced on 3^rd^ July 2023 (vertical dashed line). Means represent canopy temperature averaged across three canopy levels: top, middle, and bottom, (n = 5) ± 95% CIs. Means with asterisks above were significantly affected by treatment according to main effects analysis grouped by pot size, cultivar, and sample date with a Bonferroni p-value adjustment (* = *p*< 0.05, ** = *p*< 0.01, *** = *p*< 0.001).

Canopy temperature was significantly affected by treatment (*p*< 0.001), pot size (*p*< 0.001), cultivar (*p* = 0.001), and sample date (*p*< 0.001) and there was a significant (*p* = 0.006) four-way interaction between cultivar, pot size, treatment, and sample date (**Table 3**). When this interaction effect was broken down by cultivar, there was a significant (*p*< 0.001) three-way interaction between pot size, sample date, and treatment in both Maris Piper and Charlotte. When each cultivar was grouped by pot size, there was a significant (*p*< 0.001) interaction between treatment and sample date in all four groups. Significant effects of treatment were only seen on specific sample dates, which varied between the groups of cultivar and pot size (**Figures 3**, **4**).

**Table 3:** Main effects and interaction terms of four-way repeated measures ANOVAs, with GG corrections, for canopy temperature (°C) and average canopy SPAD of two potato cultivars (Maris Piper and Charlotte), grown in one of two pots sizes (5 and 20 L) and subjected to every other day, daily, or twice daily irrigation treatments.

Effect	Canopy Temperature (°C)				Average Canopy SPAD			
	DFn	DFd	F	p	DFn	DFd	F	p
Treatment (T)	2	48	50.5	**0.000**	2	48	0.5	0.612
Pot Size (PS)	1	48	71.9	**0.000**	1	48	0.5	0.502
Cultivar (C)	1	48	13.5	**0.001**	1	48	84.6	**0.000**
Sample Date (SD)	8.79	421.68	481.6	**0.000**	11.81	566.98	106.0	**0.000**
T x PS	2	48	13.1	**0.000**	2	48	0.0	0.952
T x C	2	48	0.2	0.795	2	48	1.2	0.306
PS x C	1	48	0.0	0.942	1	48	15.7	**0.000**
T x SD	17.57	421.68	10.1	**0.000**	23.62	566.98	1.3	0.188
PS x SD	8.79	421.68	20.3	**0.000**	11.81	566.98	8.4	**0.000**
C x SD	8.79	421.68	7.7	**0.000**	11.81	566.98	3.0	**0.001**
T x PS x C	2	48	0.2	0.786	2	48	1.6	0.220
T x PS x SD	17.57	421.68	8.9	**0.000**	23.62	566.98	0.8	0.743
T x C x SD	17.57	421.68	2.4	**0.001**	23.62	566.98	1.0	0.422
PS x C x SD	8.79	421.68	8.1	**0.000**	11.81	566.98	2.9	**0.001**
T x PS x C x SD	17.57	421.68	2.1	**0.006**	23.62	566.98	0.8	0.779

Temperature and SPAD values were sampled between 27^th^ June and 4^th^ August with a handheld laser thermometer and SPAD meter, respectively. Significant p-values (< 0.05) are indicated in bold.

**Figure 5:**
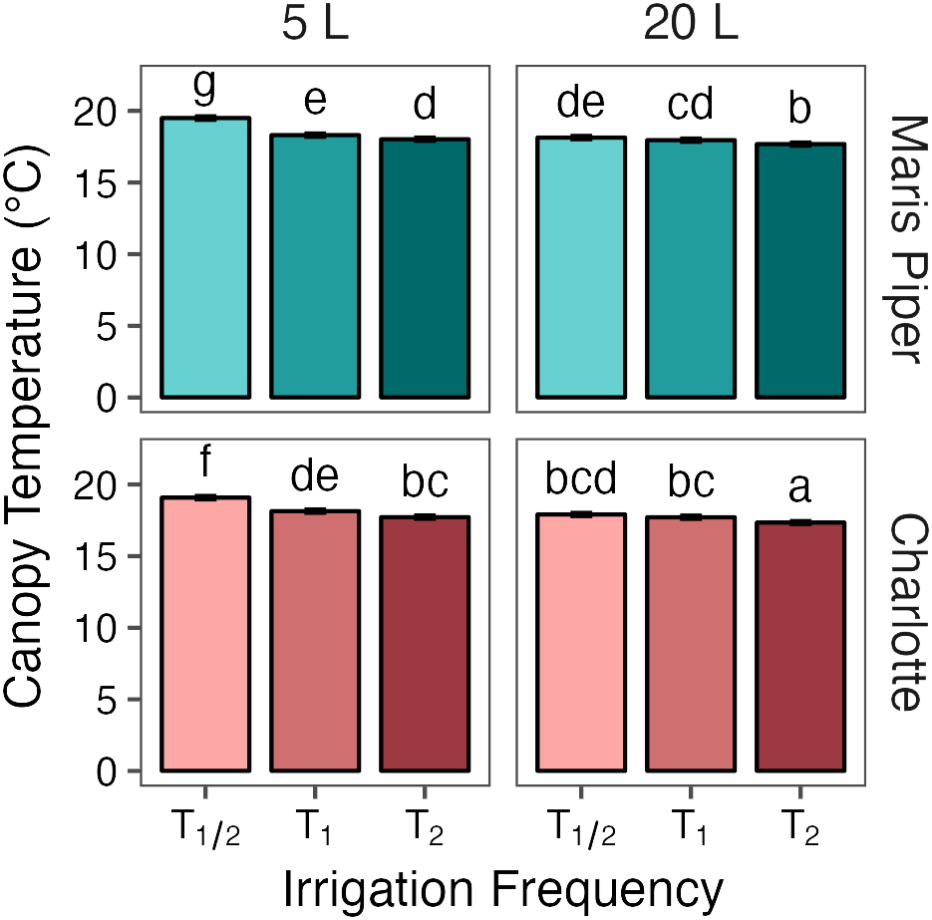
Mean canopy temperatures of two potato cultivars, Maris Piper (top facets) and Charlotte (bottom facets), grown in two pot sizes, 5 (left facets) and 20 L (right facets), each under three different water treatments: watered to capacity every other day (T_1/2_), daily (T_1_), and twice daily (T_2_). Plants were grown under an open-ended polytunnel between 1^st^ June and 4^th^ August 2023. Canopy temperature was measured between 27^th^ June and 4^th^ August. The different irrigation frequency treatments commenced on 3^rd^ July 2023 (vertical dashed line). Means represent canopy temperature averaged across three canopy levels: top, middle, and bottom, and twenty-two sample dates (n = 110) ± 95% CIs. Means with different letters were significantly different by Tukey’s test (*p*< 0.05).

Across all other factors, canopy temperature demonstrated a dose-dependent response to treatment (**Figure 5**), as each increase in irrigation frequency was associated with a significant decrease in canopy temperature (*p*< 0.05). Plants under T_1/2_ $\left(\bar{x}=18.7{}^{\circ}C\right)$ were 0.6°C warmer than those under T_1_ $\left(\bar{x}=18.0{}^{\circ}C\right)$ . Plants under T_1_ were also 0.3°C warmer than those under T_2_ $\left(\bar{x}=17.7{}^{\circ}C\right)$ . This relationship was consistent within each pot size, although canopy temperatures within each treatment were significantly higher in 5 L pots compared to 20 L pots (*p*< 0.05).

Plants under T_1/2_ in 5 L pots $\left(\bar{x}=19.3{}^{\circ}C\right)$ were 1.3°C warmer than those under the same conditions in 20 L pots $\left(\bar{x}=18.0{}^{\circ}C\right)$ . Plants under both T_1_ and T_2_ were both 0.4°C warmer in 5 L pots ( $\bar{x}=18.2{}^{\circ}C\text{ and }17.9{}^{\circ}C$ , respectively) compared to 20 L pots ( $\bar{x}=17.8{}^{\circ}C\text{ and }17.5{}^{\circ}C$ , respectively). Canopy temperatures in each group of pot size and treatment were significantly different from all other groups (*p*< 0.05), except for T_1/2_ in 20 L pots and T_1_ in 5 L pots.

This relationship between canopy temperature, treatment, and pot size was similar between the cultivars. There was a larger difference in canopy temperature between plants under T_1_ and T_1/2_ in 5 L pots compared to 20 L pots, in both Maris Piper ( $\Delta \bar{x}=+1.2{}^{\circ}C\text{ and}+0.2{}^{\circ}C$ , respectively) and Charlotte ( $\Delta \bar{x}=+0.9{}^{\circ}C\text{ and}+0.2{}^{\circ}C$ , respectively). The temperature differences between plants under T_2_ and T_1_ were more consistent, with a 0.3°C increase in canopy temperature in Maris Piper and a 0.4°C increase in temperature in Charlotte, both regardless of pot size.

The relationships between canopy temperature, treatment, pot size, and cultivar were particularly evident when grouped by sample date (**Figures 3**, **4**). The difference in canopy temperature between plants under T_1/2_ and T_1_ was frequently much larger, and more likely to be significant, in 5 L pots than 20 L pots, regardless of cultivars.

In summary, canopy temperature was significantly affected by treatment, pot size, cultivar, and sample date, with a significant interaction effect between all four factors (**Table 3**). Across all other factors, canopy temperature demonstrated a dose-dependent response to treatment, as each increase in irrigation frequency was associated with a significant decrease in canopy temperature (**Figure 5**). This relationship was consistent within each pot size, although canopy temperatures within each treatment were significantly higher in the smaller pots. All these groups were significantly different from one another, except for plants irrigated every other day in 20 L pots and every day in 5 L pots. When grouped by sample date, the difference in canopy temperature between plants irrigated every other day and daily was more likely to be significant in the smaller pots; this effect was consistent between the cultivars.

#### 3.2.4 Average canopy SPAD values were not affected by pot size or irrigation frequency

**Figure 6:**
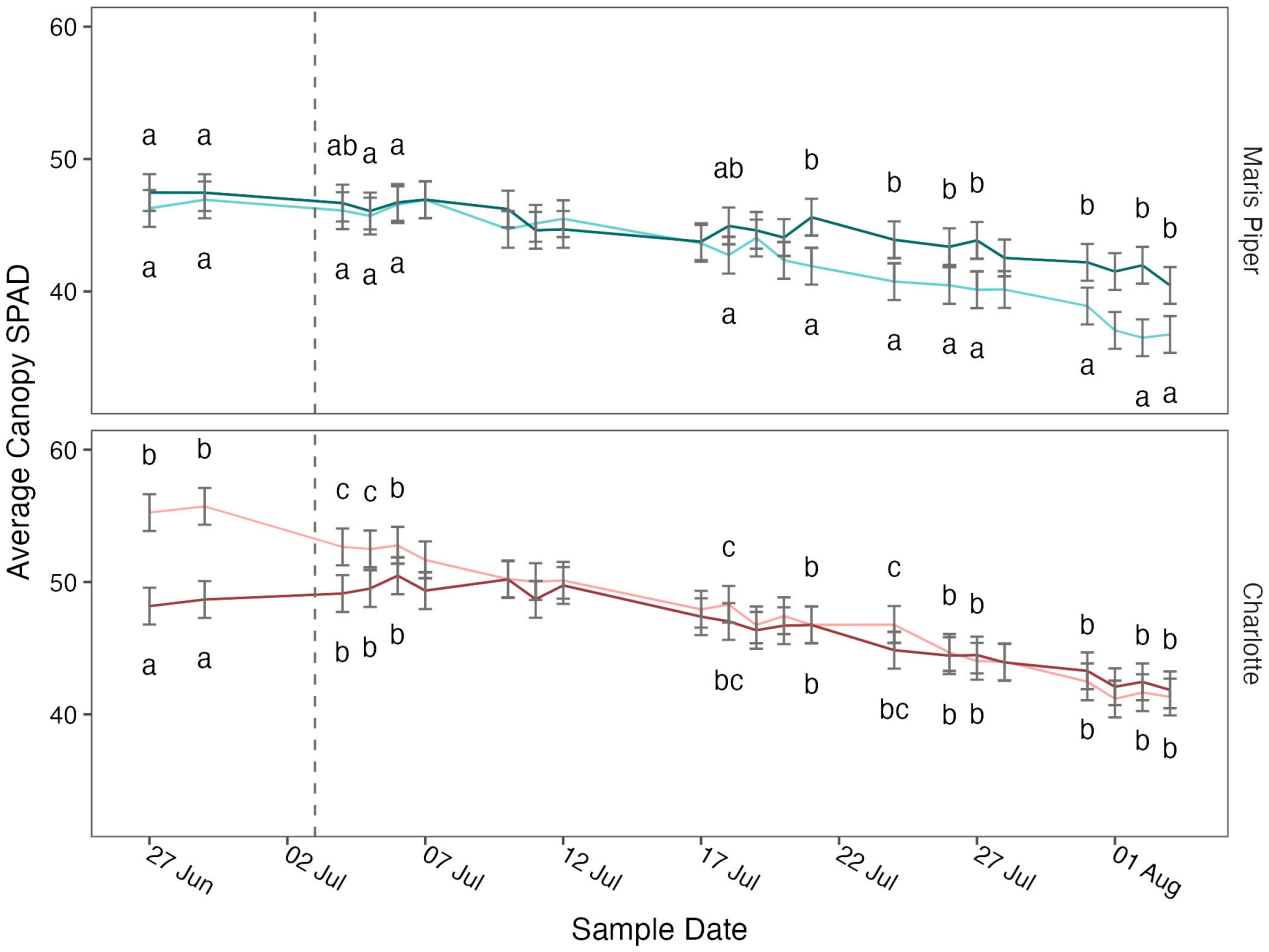
Mean canopy SPAD values of two cultivars of potato, Maris Piper (top facet) and Charlotte (bottom facet) over time, grown in two pot sizes, 5 (light lines) and 20 L (dark lines), across three water treatments: watered to capacity every other day, daily, and twice daily. Plants were grown under an open-ended polytunnel between 1^st^ of June and 4^th^ of August 2023. SPAD values were measured between 27^th^ June and 4^th^ August. The different irrigation frequency treatments commenced on 3^rd^ July 2023 (vertical dashed line). Means represent average SPAD values measured at three canopy levels per plant: top, middle, and bottom, (n = 5) ± 95% CIs. Compact letters were removed for sample dates with an insignificant (p > 0.05) interaction between pot size and cultivar. Within each sample date, means with different letters were significantly different by Tukey’s test (*p*< 0.05).

Average canopy SPAD was significantly affected by cultivar (*p*< 0.001) and sample date (*p*< 0.001) but not by pot size (*p* = 0.502) or treatment (*p* = 0.612) (**Table 3**, **Supplementary Table S1**). The four-way interaction was not significant (*p* = 0.779), but there was a significant interaction between pot size, cultivar, and sample date (*p* = 0.001). When grouped by sample date, there were significant (*p*< 0.05) interactions between pot size and cultivar on thirteen of the twenty-two sample dates. Post-hoc pairwise comparisons for each sample date with a significant interactions demonstrated that Charlotte had consistently higher SPAD values in both pot sizes, Charlotte in 5 L pots began the experiment with significantly (*p*< 0.05) higher SPAD values than Charlotte in 10 L pots and Maris Piper in both pot sizes, and Maris Piper ended the experiment with significantly (*p*< 0.05) lower SPAD values than the other three groups (**Figure 6**).

### 3.3 PlantEye measurements

#### 3.3.1 Irrigation frequency had a significant effect on digital canopy biomass in both pot sizes, but only in Maris Piper

**Figure 7:**
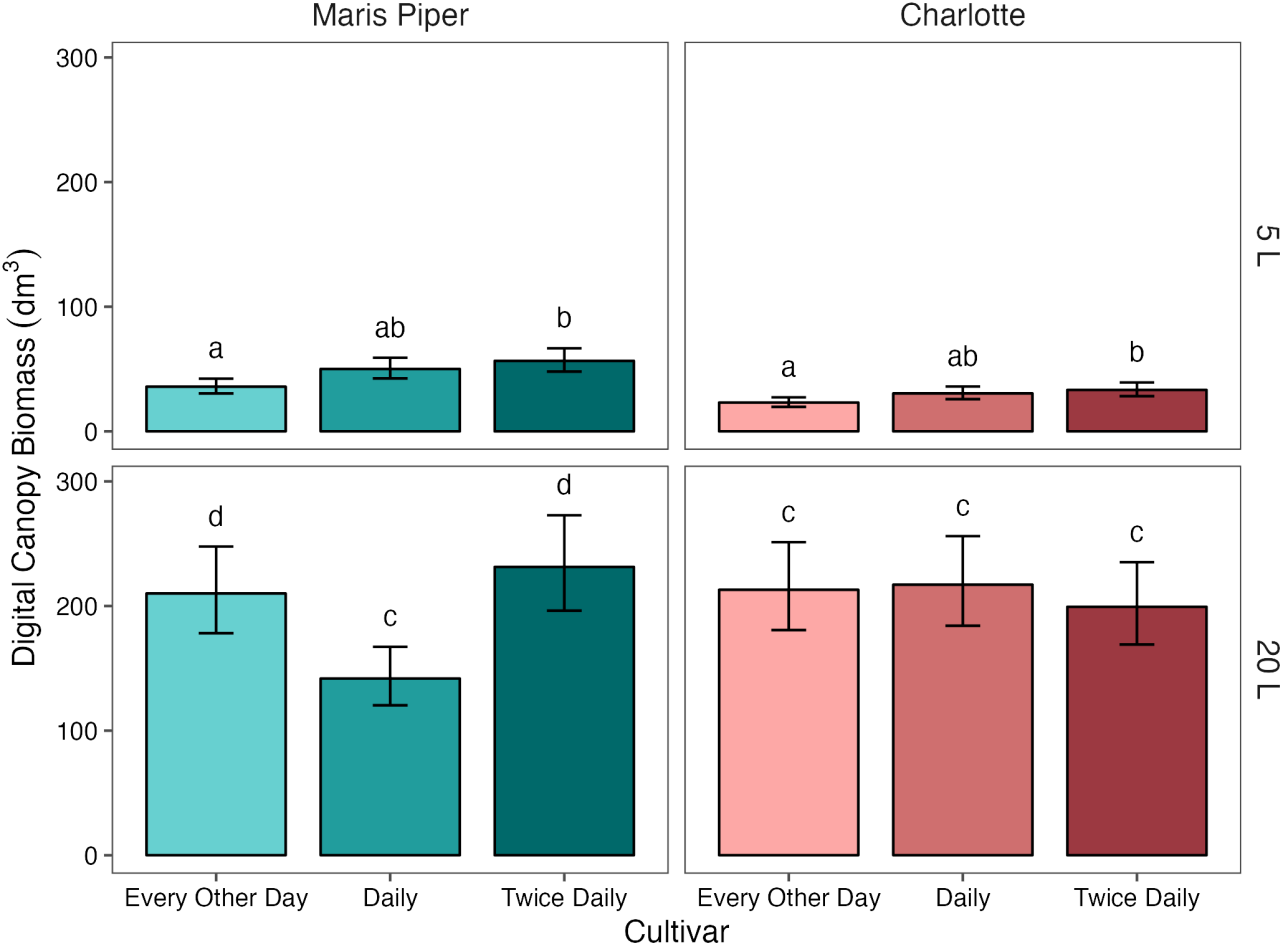
Mean digital canopy biomass of two cultivars of potato, Maris Piper and Charlotte, grown in two pot sizes, 5 and 20 L, each under three water treatments: watered to capacity every other day, daily, and twice daily. Plants were grown under an open-ended polytunnel between 1^st^ June and 4^th^ August 2023. Canopies were scanned on 13^th^ July, 42 days after planting. Means represent digital biomass, measured by HortControl (Phenospex, Heerlen, Netherlands), in decilitres cubed (n = 5) ± 95% CIs. Means with different letters within each cultivar were significantly different by Tukey’s test (*p*< 0.05).

Digital canopy biomass (**Figure 7**) was also significantly affected by pot size (*p*< 0.001), cultivar (*p*< 0.001), and treatment (*p* = 0.003). In contrast to fresh canopy biomass, there was also a marginally significant interaction between all three grouping factors (*p* = 0.05), and significant interactions between each pair of factors (**Table 4**, **Supplementary Table S1**). Again, pot size had the greatest effect, with a difference of 163.23 dm^3^ (138.1%) in digital biomass between 5 L $\left(\bar{x}=36.60\;dm^{3}\right)$ and 20 L pots $\left(\bar{x}=199.83\;dm^{3}\right)$ .

**Table 4:** Main effects and interaction terms of a three-way ANOVA (analysis of variance) for digital canopy biomass average greenness, average hue, average NDVI, average PSRI, leaf angle, light penetration depth of two potato cultivars (Maris Piper and Charlotte), grown in one of two pots sizes (5 and 20 L) and subjected to every other day, daily, or twice daily irrigation treatments.

Effect	DF	Digital Canopy Biomass (log10(dm^3^))		Average Greenness		Average Hue		Average NDVI	
		F	p	F	P	F	P	F	P
Treatment (T)	24	7.5	**0.003**	0.1	0.896	10.3	**0.001**	11.2	**0.000**
Pot Size (PS)	24	1356.7	**0.000**	225.2	**0.000**	121.3	**0.000**	296.4	**0.000**
Cultivar (C)	24	18.0	**0.000**	14.9	**0.001**	50.0	**0.000**	1.2	0.280
T x PS	24	10.6	**0.001**	8.3	**0.002**	14.4	**0.000**	2.7	0.085
T x C	24	3.6	**0.041**	23.9	**0.000**	2.4	0.115	18.4	**0.000**
PS x C	24	40.2	**0.000**	43.1	**0.000**	41.1	**0.000**	4.4	**0.047**
C x PS x T	24	3.4	**0.050**	16.1	**0.000**	8.5	**0.002**	19.1	**0.000**
Effect	DF	Average PSRI		Leaf Angle (°)		Light Penetration Depth (mm)			
		F	P	F	P	F		P	
Treatment (T)	24	915545.6	**0.000**	0.7	0.524	5.3		**0.013**	
Pot Size (PS)	24	10753737.9	**0.000**	0.7	0.422	67.9		**0.000**	
Cultivar (C)	24	4427915.0	**0.000**	2.9	0.100	1.9		0.179	
T x PS	24	1279031.5	**0.000**	1.7	0.206	1.5		0.235	
T x C	24	210004.8	**0.000**	0.3	0.717	1.3		0.282	
PS x C	24	3646813.9	**0.000**	1.0	0.335	12.9		**0.001**	
C x PS x T	24	751822.7	**0.000**	0.8	0.477	0.5		0.585	

Data were collected on 13^th^ July 2023 with two PlantEye F500 multispectral 3D scanners (Phenospex, Heerlen, Netherlands), and were processed by HortControl (Phenospex, Heerlen, Netherlands). Significant p-values (< 0.05) are indicated in bold.

When grouped by cultivar, there was a significant interaction between pot size and treatment on digital canopy biomass in Maris Piper (*p* = 0.003) but not in Charlotte (*p* = 0.246). When grouped further by pot size, treatment had a significant effect on Maris Piper in both 5 L (*p* = 0.004) and 20 L pots (*p* = 0.002). There was no interaction between pot size and treatment in Charlotte as the effect of treatment on digital canopy biomass was insignificant in 20 L pots (*p* = 1.000).

#### 3.3.2 Digital biomass was significantly correlated with fresh canopy biomass, but only in smaller pots

**Figure 8:**
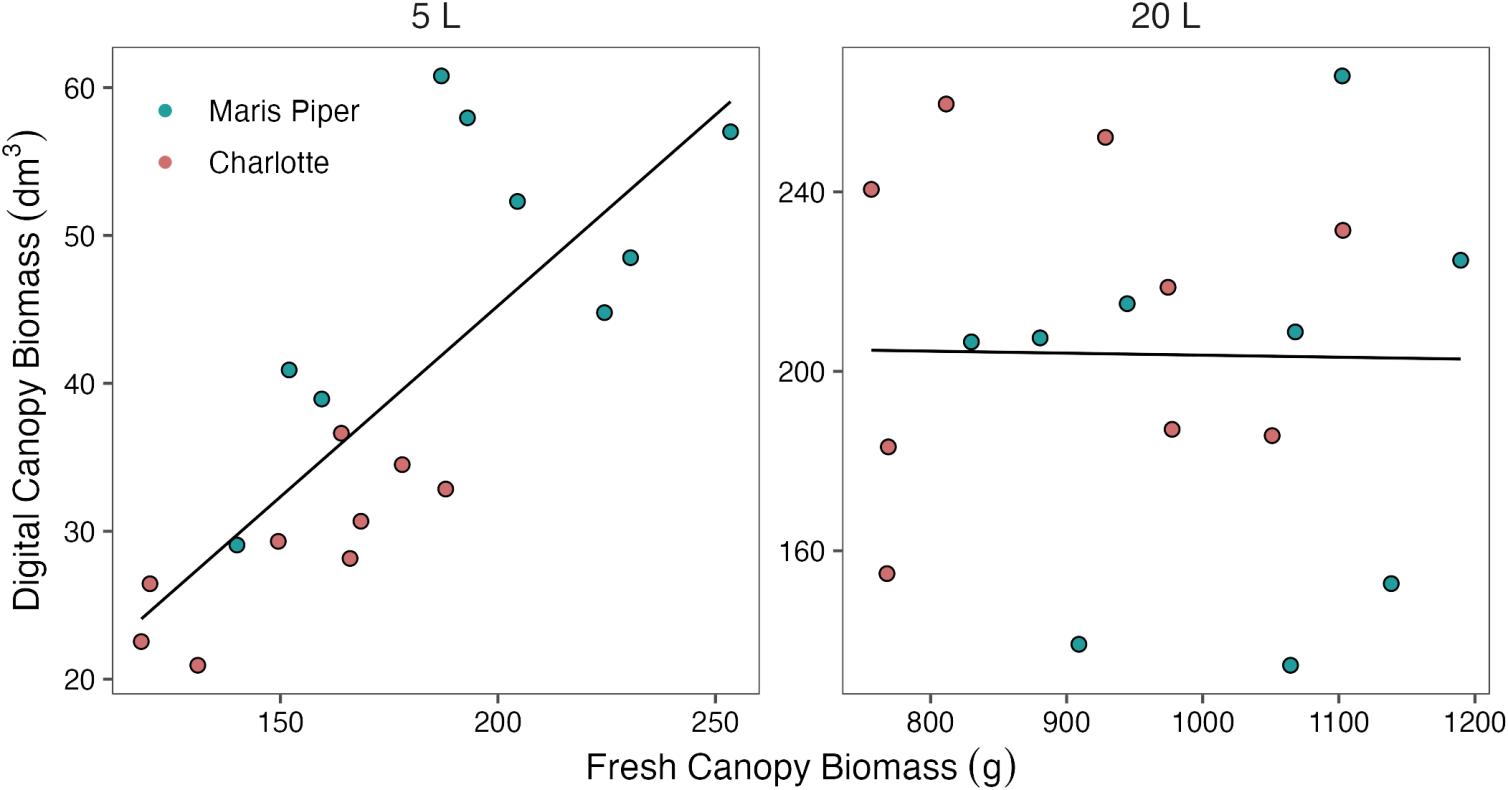
Correlations between digital canopy biomass and fresh canopy biomass for two cultivars of potato, Maris Piper (green) and Charlotte (pink), in two pot sizes, 5 L (left panel; *r* (16) = 0.780, *p*< 0.001) and 20 L (right panel; *r* (16) = 0.015, *p* = 0.952), under three water treatments: watered to capacity every other day, daily, and twice daily. Plants were grown under an open-ended polytunnel between 1^st^ June and 4^th^ August 2023. Digital canopy biomass was measured by HortControl (Phenospex, Heerlen, Netherlands) on 13^th^ of July, 42 days after planting, and canopies were harvested and weighed on 4^th^ of August, 64 days after planting.

In 5 L pots, there was a significant, strong, positive correlation between fresh canopy biomass and digital canopy biomass (*r* (16) = 0.780, *p*< 0.001). However, in 20 L pots, this correlation was not significant (*r* (16) = 0.015, *p* = 0.952) (**Figure 8**). A similar correlation was found between manual and digital measurements of plant height. In 5 L pots there was a significant positive correlation (*r* (16) = 0.896, *p*< 0.001), but in 20 L pots, this correlation was not significant, (*r* (16) = -0.390, *p* = 0.110).

#### 3.3.3 Average greenness was significantly correlated with SPAD values, but only in smaller pots

Average greenness was significantly affected by pot size (*p*< 0.001) and cultivar (*p* = 0.001), but not by treatment (*p* = 0.896) (**Table 4**). There was also a significant three-way interaction between all three grouping factors (*p*< 0.001). When grouped by cultivar, there was a significant interaction between pot size and treatment in Charlotte (*p*< 0.001) but not Maris Piper (*p* = 0.204). The effect of treatment on Charlotte was only significant in 20 L pots (*p*< 0.001). Within this group, average greenness was significantly (*p*< 0.05) higher with under T_1/2_ compared to T_1_ and T_2_, which were not significant different from each other. Overall, Maris Piper $\left(\bar{x}=0.33\right)$ had a slightly (0.04 index units, 4.5%) higher average greenness than Charlotte $\left(\bar{x}=0.31\right)$ and plants in 20 L pots $\left(\bar{x}=0.35\right)$ had a higher (0.06, 17.7%) average greenness than those in 5 L pots $\left(\bar{x}=0.29\right)$ . Overall, there was no significant correlation between average greenness and average canopy SPAD values (*r* (16) = -0.213, *p* = 0.213). When broken down by pot size, there was a significant, very strong, negative correlation between these two variables in 5 L pots (*r* (16) = -0.864, *p*< 0.001), but not in 20 L pots (*r* (16) = 0.344, *p* = 0.162).

#### 3.3.4 Irrigation had significant effects on average hue, but only for Charlotte in small pots and Maris Piper in large pots

Average hue was significantly affected by all three grouping factors (pot size, *p*< 0.001; cultivar, *p*< 0.001; treatment, *p* = 0.001) and there was a significant three-way interaction (*p* = 0.002) (**Table 4**). When grouped by cultivar, there were significant interactions between pot size and treatment in both Maris Piper (*p*< 0.001) and Charlotte (*p* = 0.012). Treatment had a significant effect on Charlotte in 5 L (*p* = 0.008) and on Maris Piper in 20 L pots (*p*< 0.001), but not on Charlotte in 20 L pots (*p* = 0.788) or on Maris Piper in 5 L pots (*p* = 0.696). However, the differences in average hue between treatments within these groups were very small (≤ 5.3%).

#### 3.3.5 Irrigation frequency significantly affected average NDVI, but only in large pots

Whole-plant average NDVI was significantly affected by treatment (*p*< 0.001) and pot size (*p*< 0.001), but not by cultivar (*p* = 0.280) (**Table 4**). There was a significant three-way interaction between all three grouping factors (*p*< 0.001). When grouped by cultivar, there were significant two-way interactions between treatment and pot size in both Maris Piper (*p* = 0.048) and Charlotte (*p*< 0.001). Within each cultivar, the effect of treatment was only significant in 20 L pots (Maris Piper, *p*< 0.001; Charlotte, *p*< 0.001). However, the percentage differences between treatments within these groups were small (< 5%).

#### 3.3.6 Average PSRI was consistently affected by irrigation frequency in both pot sizes and cultivars

Whole-plant average PSRI was significantly affected by treatment (*p* = 0.001), pot size (*p*< 0.001), and cultivar (*p*< 0.001) and there was a significant three-way interaction between all three grouping factors (*p* = 0.002) (**Table 4**). When grouped by cultivar, there were significant interactions between treatment and pot size in both Maris Piper (*p*< 0.001) and Charlotte (*p*< 0.001). Within each cultivar, the effect of treatment was significant in both cultivars in both pot sizes (Maris Piper: 5 L, *p*< 0.001; 20 L, *p*< 0.001; Charlotte: 5 L, *p*< 0.001; 20 L, *p*< 0.001). The percentage differences between these groups were large, but the absolute differences between significantly different groups were still small (< 0.03 index units).

#### 3.3.7 Leaf angle was not affected by irrigation frequency, pot size, or cultivar

Leaf angle was not significantly affected by any of the grouping factors and there were no significant interactions (**Table 4**).

#### 3.3.8 Light penetration depth ranking of each cultivar was different between the pot sizes

Light penetration depth was significantly affected by treatment (*p* = 0.013) and pot size (*p*< 0.001), but not by cultivar (p = 0.179) (**Table 4**). There was a single significant interaction between pot size and cultivar (*p* = 0.001); the effect of cultivar was significant in 5 L pots (*p* = 0.004), but not in 20 L pots (*p* = 0.262). The difference in light penetration depth between Maris Piper and Charlotte was 82.49 mm (46.7%) in 5 L pots, compared to only 36.61 mm (11.7%) in 20 L pots. Light penetration depth was shorter for Charlotte in 5 L pots $\left(\bar{x}=135.52\;mm\right)$ compared to Maris Piper $\left(\bar{x}=218.01\;mm\right)$ , but longer in 20 L pots (Charlotte, $\bar{x}=331.47\;mm$ ; Maris Piper, $\bar{x}=294.86\;mm$ ). Light penetration depth also increased with increasing irrigation frequency, but the only significant difference occurred between T_1/2_ and T_2_ (*p*< 0.05).

## 4 Discussion

### 4.1 Fresh tuber yield, but not fresh canopy biomass, support the water-availability hypothesis of pot binding

#### 4.1.1 Fresh tuber yield

This experiment aimed to investigate the water availability hypothesis of pot binding in potato. The hypothesis states that pot binding, i.e., the confounding effects of small pots on plant morphophysiology, is primarily a result of an unintentional drought stress experienced by purportedly well-watered plants (Sinclair et al., 2017). Pot binding is thought to occur when the water holding capacity of a potted substrate is insufficient to prevent drought stress between irrigation periods (Turner, 2019). Previous research has suggested that pot binding can be mitigated by providing plants with 1 L of substrate for every gram of dry biomass that a plant is expected to produce (Poorter et al., 2012). As potato has been observed to generate over 1,000 g of dry biomass (Wheeler and Tibbitts, 1987), this recommendation is impractical for this crop in most controlled environmental facilities.

To test this hypothesis, we grew two cultivars of potato, Maris Piper and Charlotte, in two pot sizes, 5 and 20 L, each under one of three water treatments: irrigation to saturation twice daily (T_2_), daily (T_1_), or every other day (T_1/2_). If pot binding is a product of water unavailability under T_1_ conditions, then morphophysiological indicators of drought stress should be observed in both T_1/2_ and T_1_ plants. This effect should be mitigated by increasing the pot size (Poorter et al., 2012; Turner, 2019) or by increasing the irrigation frequency (Sinclair et al., 2017; Turner, 2019).

Therefore, we hypothesised that there would be greater similarities in traits known to be affected by drought stress between T_1_ and T_1/2_ treatments in the smaller pots. We also hypothesised that this effect would be mitigated in the larger pots, and by increasing the frequency of irrigation from T_1_ to T_2_. We assessed several morphophysiological indicators of drought stress that have previously been shown to affect potato, including tuber yield, canopy biomass, canopy and tuber dry matter (Obidiegwu et al., 2015; Hill et al., 2021), average canopy temperature (Stark et al., 1991; Ninanya et al., 2021) and SPAD values (Li et al., 2019).

Fresh tuber yield and canopy biomass are the two morphological traits most sensitive to water-restriction in potato (Jefferies and Mackerron, 1987, Jefferies and Mackerron, 1993). As plant tissue growth is primarily a result of cell elongation (Shao et al., 2009), which is driven by high turgor pressure (Lockhart, 1965), water deficits result in reduced growth of many tissues. Canopy biomass is also particularly affected by water-restriction in potato, compared to other crops. Leaf growth in most crop species ceases when the fraction of transpirable soil water drops below 40-50%; in potato leaves, growth is negligible once the available soil water reaches 60% (Weisz et al., 1994).

In this experiment, fresh tuber yield was significantly reduced by decreasing irrigation frequency from T_1_ to T_1/2_, but only in the larger, 20 L pots. In the 5 L pots, there was no meaningful difference in tuber yield between T_1_ and T_1/2_ (**Figure 1**). This is consistent with the water availability hypothesis of pot binding, as the difference in yield between the hypothetically well-watered and intentionally drought stressed plants was minor compared to the yield difference in the large pots. This also supports previous research, which found that potato yield reductions associated with water-restriction increased with pot size (Hill et al., 2023)^1^.

Increasing irrigation from T_1_ to T_2_ was not sufficient to increase fresh tuber yield in 5 L pots (**Figure 1**), suggesting that saturation twice per day in very small pots is still insufficient to prevent pot binding in potato. It could be suggested that yields in the small pots were limited by the pot volume, rather than drought stress. However, there was no evidence that this was the case as yields in the small pots were very low (296.0 g) and the tubers occupied only a small amount of the pot volume. There was also a further, albeit not significant, increase in yield between T_1_ and T_2_ in 20 L pots. This suggests that, while larger pots may prevent pot binding well enough to detect significant yield differences between T_1/2_ and T_1_, they may not eliminate it altogether under daily irrigation.

#### 4.1.2 Fresh canopy biomass

In contrast with fresh tuber yield, canopy biomass was similarly affected by water restriction in the two pot sizes, with a significant decrease in biomass between T_1_ and T_1/2_ occurring in both. This finding is inconsistent with the water availability of pot binding, as are the percentage differences in biomass between T_1_ and T_1/2_ in the two pot sizes. Canopy biomass was more effected by water-restriction in 5 L pots (27%), compared to 20 L pots (16%) (**Figure 2**). Increasing irrigation frequency from T_1_ to T_2_ had an insignificant effect on canopy biomass in both pot sizes, although it was associated with a slight increase in biomass in 5 L pots, which suggests that T_1_ might be unable to maintain maximum canopy biomass accumulation in the smaller pots.

It is not clear why fresh tuber yield and canopy biomass are affected differently by water restriction in the two pot sizes. It is possible that, due to extremely limited water availability, yield was maintained at the expense of biomass under T_1/2_ in the smaller pots. This seems unlikely as both fresh tuber yield and canopy biomass have previously been shown to decrease in 4.7 L pots from a similar treatment to T_1/2_ (irrigation to saturation every other day) to a treatment that restricts water even further (Rolando et al., 2015).

#### 4.1.3 Canopy and tuber dry matter

Both canopy and tuber dry matter percentages were primarily affected by cultivar, with pot size and treatment having small effects. Maris Piper had a significantly higher dry matter concentration than Charlotte in both the canopy and tubers (**Table 2**). Dry matter content is known to vary between potato cultivars (Navarre et al., 2009), and is related to cultivar maturation. Researchers have previously defined maturation in potato as the point of maximum dry matter accumulation (Sabba et al., 2007), which, in the absence of stress, is dependent on life cycle length. Late maturing cultivars, including Maris Piper, can delay senescence for longer than early cultivars, including Charlotte, facilitating greater radiation interception and photosynthesis over time (Aliche et al., 2019). This allows dry matter production to continue for longer in late maturing cultivars, which accounts for the differences observed here.

The canopy and tuber dry matter percentages of both cultivars were relatively unaffected by treatment (**Table 2**), with a significant but small decrease in the former with increasing irrigation frequency and no effect in the latter. Above- and below-ground dry matter accumulation responses to water-restriction are known to vary greatly between cultivars (Hill et al., 2021). However, the differences observed between the cultivars were significantly confounded by pot size, both in the canopy and tubers. The tuber dry matter of Charlotte was identical in both pot sizes, whereas that of Maris Piper was significantly higher in the smaller pots compared to the larger pots. Above ground, this was completely reversed, as the canopy dry matter content of Maris Piper being unaffected by pot size and an association between small pots and higher dry matter concentration in Charlotte. This suggests that something other than water availability is causing the confounding effects of small pots on potato morphophysiology.

Previous research in tall but narrow 11.8 L pots (⌀ = 10 cm) has shown that both self- and reciprocally grafted potato canopies elicit greater control over dry matter accumulation than root stocks (Jefferies, 1993). This, coupled with the small and non-interactive effects of water-restriction in both pot sizes, suggests that pot binding may have a confounding effect on potato canopies that is not related to inadvertent drought stress. The cause of this is beyond the scope of this experiment, but previous research with five cowpea cultivars in 11, 17, and 76 L pots, suggested that small pots were associated with greater root abscisic acid production, and downstream reductions in canopy and root biomass, even under well-watered conditions (Ismail et al., 1994). Similar findings have also been found in tomato, where shoot growth was restricted in small pots despite “great care” (Turner, 2019) to maintain consistent water and nutrient availability between pot sizes (Hurley and Rowarth, 1999).

### 4.2 Average canopy temperatures support the water-availability hypothesis and suggest pot binding can be mitigated by increasing irrigation frequency, but SPAD values suggest pot binding might also be due to root restriction

#### 4.2.1 Canopy temperature

To provide an indication of drought stress during the experiment, average canopy temperature was measured throughout. In plants, canopy temperature is kept within the lethal limits for a particular species through transpiration (Gates, 1964). As relatively cool groundwater is taken up by the roots and moved through the plants to the leaves, it absorbs the excess thermal energy generated by solar radiation from the surrounding tissue and removes it from the plant by evaporating through the stomata (Lin et al., 2017). Even tiny amounts of transpiration can dissipate significant amounts of thermal energy and cool plant canopies by a few degrees (Gates, 1964).

Measuring potato canopy temperatures under well-watered conditions was evaluated as a method of evaluating drought tolerance between cultivars over 30 years ago (Stark et al., 1991). The method was successful in potato and other crops, as canopy temperature and water use are negatively correlated under well-watered conditions (Keener and Kircher, 1983; Chaudhuri and Kanemasu, 1985), and canopy temperature and drought susceptibility are positively correlated under water-restricted conditions (Blum et al., 1989; Stark et al., 1991).

With recent advancements in remote sensing, ground and aerial measurements of canopy temperature have been investigated as methods of estimating drought stress in potato (Rud et al., 2014). Previous research has demonstrated that canopy temperature can be integrated within a water stress index that is strongly correlated with stomatal conductance (Rud et al., 2014). This index was also shown to increase under water-restricted conditions compared to well-watered controls (Rud et al., 2014).

Across this experiment, each increase in irrigation frequency was associated with a significant decrease in canopy temperature (**Figure 5**). The differences between the treatments were smaller than previously suggested (Gates, 1964), varying by ~1°C above or below the daily irrigation treatment (T_1_). This is related to climatic conditions, as the mean ambient air temperature at 09:00, one hour before canopy temperatures were measured, was only 17.8 ± 0.5°C. Within each treatment, canopy temperature was consistently higher in the smaller pots than in the larger pots (**Figure 5**). Again, the differences were small (< 1.5°C) due to the low potential evapotranspiration early in the photoperiod.

The difference between T_1_ and T_1/2_ was significantly larger in the smaller pots compared to the larger pots (**Figure 5**). This seems contrary to the prediction of the water availability hypothesis of pot binding. If pot binding was a result of the relative inability of daily irrigation to maintain potential evapotranspiration in small pots, then canopy temperatures should be more similar between T_1_ and T_1/2_ in smaller pots than in larger pots. However, the high canopy temperatures under T_1/2_ conditions in 5 L pots shows that this treatment produces much more severe drought stress than the same treatment in 20 L pots. Importantly, canopy temperatures decreased relative to T_1_ under T_2_ in both pot sizes, suggesting that neither pot size could sustain potential evapotranspiration under T_1_ conditions.

The only combinations of pot size and treatment that were not significantly different from each other were T_2_ in 5 L pots and T_1/2_ in 20 L pots. This shows that the effects of pot binding do result from water unavailability can be mitigated to some extent by increasing irrigation frequency. However, canopy temperatures under T_1/2_ conditions in 20 L pots were still significantly higher than under T_1_ and T_2_ conditions, which demonstrates that maintaining adequate water availability for maximum transpiration is not possible in 5 L pots with twice daily watering to saturation.

Canopy temperatures in both cultivars responded similarly to each treatment, with Maris Piper being significantly warmer under each (**Figure 5**). This is a result of Maris Piper being a later maturing cultivar than Charlotte. Late cultivars produce larger canopies (Hill et al., 2021) and thus require greater volumes of water to maintain potential transpiration (Fandika et al., 2016). The canopy temperature of Maris Piper was also more affected by water-restriction from T_1_ to T_1/2_ than Charlotte in 5 L pots (+1.2°C versus +0.9°C), but similarly affected by water-restriction in 20 L pots (+0.2°C in both). This highlights the necessity of considering cultivar specific water requirements when selecting experimental pot sizes to prevent pot binding in potato.

When broken down by sample date, canopy temperature was affected by treatment more frequently in the 5 L pots (**Figure 3**, **Figure 4**). Although the differences across the whole experiment were typically significant, the effects of treatment on canopy temperature were only significant on 3 or 4 days in 20 L pots in Maris Piper and Charlotte, respectively. This contrasts with the 9 and 6 days where treatment had a significant effect on canopy temperature in 5 L pots in same cultivars, respectively. This is indicative of the extreme drought stress experienced by plants under T_1/2_ conditions in 5 L pots, which have previously been used as a well-watered control condition (Li et al., 2019; Hill et al., 2023)^1^.

As the differences in canopy temperatures between treatments were significant across the experiment, it is likely that significant differences on individual sample dates may have been more frequent with greater sample sizes. This demonstrates the potential utility of canopy temperature as a metric by which potato irrigation systems can be controlled. If slight differences between canopy temperatures in the field and a concurrent or historical well-watered population can be detected, then irrigation could be scheduled when canopy temperatures begin to rise.

#### 4.2.2 Average SPAD values

SPAD meter readings are a reliable proxy for chlorophyll content (Borhan et al., 2017), and have been shown to be very strongly correlated with chlorophyll content in wheat, rice, and soybean, R^2 ^= 0.93 (Monje and Bugbee, 1992); and *Arabidopsis thaliana*, R^2 ^= 0.98 (Ling et al., 2011). In potato, SPAD values have been closely approximated with a computer imaging technique (Borhan et al., 2017), demonstrating the possibility of crop water- and nutrient-management with remote measures of canopy greenness.

Potato SPAD values have previously been shown to increase due to water-restriction (Ramírez et al., 2014; Rolando et al., 2015; Rudack et al., 2017; Li et al., 2019), probably due to decreasing leaf water contents increasing chlorophyll concentrations (Rolando et al., 2015; Gervais et al., 2021). However, more recent work has shown that the effects of water deficits on chlorophyll content in potato varies greatly depending on cultivar and growth stage (Mthembu et al., 2022).

For example, chlorophyll content in the *Solanum tuberosum* cv. Panamera increased with water-restriction during tuber initiation but decreased with water-restriction in the vegetative, tuber bulking, and maturation stages. In contrast, chlorophyll content in the cv. Bikini increased under water-restriction in every growth stage other than tuber initiation (Mthembu et al., 2022). This variability may explain why no effect of treatment was observed here, with no interactions between treatment or any other grouping factor.

Average canopy SPAD was affected by an interaction between cultivar, pot size, and sample date (**Table 3**). Charlotte was greener than Maris Piper, demonstrating the variability in SPAD values between cultivars, regardless of treatment, previously observed (Mthembu et al., 2022). It is unclear why Charlotte in 5 L pots had significantly higher SPAD values than Charlotte in 20 L pots at the beginning of sampling. However, after 9 days the difference between pot sizes in Charlotte had disappeared, and SPAD values in both pot sizes remained similar for the duration of sampling (**Figure 6**).

In Maris Piper, average SPAD values decreased at a faster rate in the larger pots (**Figure 6**). This was associated with the faster rate of senescence observed with Maris Piper in the 5 L pots, an effect that has been observed before (Hill et al., 2023)^1^. It is unlikely that early senescence in Maris Piper is a result of water unavailability in smaller pots, as senescence did not occur at a faster rate in the water-restricted plants. Instead, it is possible that the early onset of senescence was a product of nutrient unavailability, another proposed cause of pot binding (Poorter et al., 2012).

Root volume restriction in aerated liquid culture has previously been observed to reduce chlorophyll content and cause early senescence in alder (*Alnus glutinosa*) seedings (Tschaplinski and Blake, 1985) reduced leaf water potential due to an imbalanced root/shoot ratio (Tschaplinski and Blake, 1985). Research with starfruit (*Averrhoa carambola*) has also shown reduced leaf water potential and photosynthetic rate with root restriction, but this effect was compounded by water-restriction (Ismail and Noor, 1996). Root restriction also increased the rate of maturation in starfruit.

If an imbalanced root/shoot ratio is a component of pot binding, then increasing irrigation frequency to mitigate water unavailability will be limited in its capacity to alleviate pot binding in small pots. As average canopy SPAD values were unaffected by treatment in this experiment, it is possible that chlorophyll content was more affected by root restriction than water-unavailability. This would explain why Maris Piper was more affected than Charlotte here, as the former produced larger canopies in both pot sizes. However, as neither leaf water potential or root/shoot ratios were measured here, further research is needed to assess the relative effects of root- and water-restriction on leaf chlorophyll content in potato.

### 4.3 Digital phenotyping is less valid for larger plants with high self-shading

In this study, digital phenotyping tools (PlantEye F500 & HortControl) were used to measure canopy biomass and height. The measurements produced with these tools were compared to manual measurements of canopy biomass and plant height, which are established methods of assessing the effects of water-restriction in potato (Elsayed et al., 2021; Ninanya et al., 2021; Mthembu et al., 2022). There was a significant positive, correlation between digital and manual measures of both canopy biomass (**Figure 8**) and height, but only in 5 L pots. In the 20 L pots, there was no clear relationship between digital and manual measurements of canopy biomass or plant height.

Previous experiments have found positive correlations between PlantEye measurements of leaf area and manually collected reference measurements, including in soybean, R^2 ^=^ ^0.89 to 0.91 (Manavalan et al., 2021); peanut, R^2 ^=^ ^0.94; cowpea, R^2 ^=^ ^0.93; and pearl millet, R^2 ^=^ ^0.86 (Vadez et al., 2015). However, these studies focussed on early plant growth to maximise the sample size. The authors suggested that overlapping leaves may result in inaccurate measurements of leaf area and digital biomass for more mature plants, or crops with high leaf area indices (Vadez et al., 2015; Manavalan et al., 2021).

Here, the plants were scanned at 42 DAP, by which time those in 20 L pots may have exceeded the threshold leaf area index (LAI) of 1.5, above which digital phenotyping of leaf area and biomass becomes increasingly inaccurate (Vadez et al., 2015). This cannot be confirmed as LAI was not measured manually and the digital measurements are invalid, at least in 20 L pots. Leaf overlap, or self-shading, is known to occur in leaf-type cultivars of potato (Schittenhelm et al., 2006) and explains the discrepancy in accuracy of digital biomass measurements between the pot sizes found here and previously (Hill et al., 2023)^1^. Further research is needed to define more accurate LAI thresholds for valid digital phenotyping of morphological traits in potato and other crops. However, digital phenotyping of canopy biomass and plant height, at least with the platform used here, is inaccurate in mature potato plants in pots ≥ 20 L.

Leaf angle was also measured in this study with digital phenotyping tools, and was found to be unaffected by treatment, pot size, and cultivar, with no significant interactions (**Table 4**). This contradicts previous research, which has suggested leaf angle is a secondary trait with potential as an indicator of drought tolerance under water-restricted conditions (Mulugeta Aneley et al., 2023). In potato, leaf angle has been shown to be ~5° higher in water-restricted plants than control plants during the day light period, with treatment having a significant effect on leaf movement: an integration of leaf angle throughout the diurnal cycle (Mulugeta Aneley et al., 2023). A positive correlation between leaf angle and the independently verified drought tolerance of twenty potato cultivars has also been observed (Köhl et al., 2023).

Similar results have been found in wheat (Lonbani and Arzani, 2011) and soybean (Martynenko et al., 2016). It’s possible that the high degree of self-shading present in potato confounded the measurements of leaf angle by the PlantEye, as is the case with leaf area and digital biomass (Vadez et al., 2015; Manavalan et al., 2021). However, it should be noted that the significant effect of treatment on leaf movement in potato was observed with a previous model (F400) of PlantEye (Mulugeta Aneley et al., 2023). This experiment was conducted in 30 L pots, suggesting pot size may also have a confounding on leaf angle in potato. However, in the absence of a direct comparison between plants in 20 and 30 L pots, or with field-grown plants, the cause of the null result observed here remains unclear.

## 5 Conclusion

We investigated the water availability hypothesis of pot binding, i.e., the confounding effects of small pots on plant morphophysiology, in potato. We assessed whether these effects could be mitigated in practical pot sizes for high-throughput phenotyping platforms by reducing the inter-irrigation period. The validity of digital measurements of plant morphology were also assessed by comparison with established, low-tech methods.

The analysis of fresh tuber yield, but not fresh canopy biomass, supported the water availability hypothesis of pot binding. Increasing irrigation frequency from every other day to daily was only associated with a significant increase in fresh tuber yield in the larger pots, suggesting a similar intensity of drought stress under both treatments in the smaller pots. Further increasing the irrigation frequency from daily to twice daily was insufficient to significantly increase fresh tuber yields in both pot sizes but did cause an insignificant increase in fresh tuber yield in the large pots, suggesting daily irrigation might not be sufficient to completely prevent pot binding even in larger pot sizes.

Canopy biomass appeared to be less affected by pot binding as reducing irrigation from daily to every other day significantly reduced biomass in both pot sizes. There was a small increase in biomass when the irrigation frequency was increased to twice daily in the small pots, but this was not significant and therefore does not strongly support the water availability hypothesis.

Canopy temperatures were significantly higher in the small pots under each irrigation frequency, which strongly supports the water availability hypothesis as higher canopy temperature is a reliable indicator of drought stress in potatoes. The canopy temperatures of Maris Piper, a late maturing cultivar, were more affected than those of the early maturing cultivar, Charlotte, in small pots, highlighting the importance of considering cultivar-specific water requirements when selecting experimental pot sizes. Canopy temperatures were similar between twice daily irrigation in small pots and irrigation every other day in large pots and were reduced in large pots with increasing irrigation frequency. This suggests that increasing irrigation frequency might be unable to prevent pot binding due to water unavailability in small pots, but increasing irrigation frequency is able to mostly mitigate pot binding in large pots. Further research is needed to define the optimum pot size and irrigation protocol to completely prevent pot binding for phenotyping experiments.

Digital phenotyping was found to be less valid for larger plants, probably due to a higher degree of self-shading. We found significant positive correlations between digital and manual measurements of canopy biomass and plant height, but only in small pots. Further research should attempt to define an appropriate leaf area index threshold for valid digital phenotyping in potato.

## Data availability statement

The datasets generated and analysed for this study can be found in the Zendo repository at https://doi.org/10.5281/zenodo.10707587.

## Author contributions

DH: Writing – review & editing, Writing – original draft, Visualization, Project administration, Methodology, Investigation, Formal analysis, Conceptualization. LC: Writing – review & editing, Investigation. DN: Writing – review & editing, Supervision, Resources. JH: Writing – review & editing, Supervision. LB: Project administration, Writing – review & editing, Supervision, Funding acquisition.

## Footnotes

1 Hill, D., Nelson, D., Hammond, J., and Bell, L. (2023). ‘Small pots confound and diminish the effects of water-restriction on potato (Solanum tuberosum L.) morphophysiology’. University of Reading.
