# Supplementary material for: Investigating the water availability hypothesis of pot binding: small pots and infrequent irrigation confound the effects of drought stress in potato (Solanum tuberosum L.)
Source: Front Plant Sci. 2024 Jun 13;15:1399250. doi: 10.3389/fpls.2024.1399250 (PMC11208687; doi:10.3389/fpls.2024.1399250)
Supplement: Supplementary file 1 [file Table_1.docx]

Supplementary Material

# Supplementary Tables

Supplementary Table 1. Mean fresh tuber yield, average tuber mass, tuber dry matter, fresh canopy biomass, canopy dry matter, digital biomass, canopy temperature, canopy SPAD values, average greenness, average hue, average NDVI, average PSRI, leaf angle, and light penetration depth of two cultivars of potato (Maris Piper and Charlotte), in two pot sizes (5 and 20 L), under three irrigation frequencies (every other day, T_1/2_; daily, T_1_; twice daily, T_2_). Canopies were harvested and weighed on 4^th^ August; tubers on 18^th^ August 2023. Fresh tuber yield, average tuber mass, tuber dry matter, fresh canopy biomass, and canopy dry matter were measured manually. Canopy temperature and SPAD values were averaged across measurements sampled between 27^th^ June and 4^th^ August. The other variables were measured with two Phenospex PlantEye F500s on 13^th^ July.

|  | Fresh Tuber Yield (g) | | | | Average Tuber Mass (g) | | | | Tuber Dry Matter (%) | | | |
| --- | --- | --- | --- | --- | --- | --- | --- | --- | --- | --- | --- | --- |
| Treatment | Maris Piper | | Charlotte | | Maris Piper | | Charlotte | | Maris Piper | | Charlotte | |
|  | 5 L | 20 L | 5 L | 20 L | 5 L | 20 L | 5 L | 20 L | 5 L | 20 L | 5 L | 20 L |
| T_1/2_ | 338.0 | 1097.8 | 274.4 | 1010.6 | 28.4 | 44.3 | 29.4 | 42.2 | 22.4% | 20.4% | 18.6% | 17.2% |
| T_1_ | 308.6 | 1208.9 | 289.9 | 1109.6 | 31.0 | 48.0 | 34.9 | 42.5 | 21.4% | 20.0% | 18.0% | 18.0% |
| T_2_ | 295.2 | 1370.9 | 272.0 | 1099.4 | 28.6 | 53.5 | 32.0 | 42.7 | 21.8% | 19.2% | 17.6% | 18.4% |
|  |  |  |  |  |  |  |  |  |  |  |  |  |
|  | Fresh Canopy Biomass (g) | | | | Canopy Dry Matter (%) | | | | Digital Biomass (dm3) | | | |
| Treatment | Maris Piper | | Charlotte | | Maris Piper | | Charlotte | | Maris Piper | | Charlotte | |
|  | 5 L | 20 L | 5 L | 20 L | 5 L | 20 L | 5 L | 20 L | 5 L | 20 L | 5 L | 20 L |
| T_1/2_ | 162.9 | 923.3 | 124.3 | 809.2 | 11.2% | 10.6% | 9.2% | 7.1% | 35.9 | 210.1 | 23.2 | 213.1 |
| T_1_ | 210.8 | 1074.3 | 166.5 | 950.2 | 10.2% | 11.2% | 8.1% | 6.6% | 50.1 | 141.9 | 30.5 | 217.2 |
| T_2_ | 214.3 | 1061.4 | 177.6 | 929.6 | 10.0% | 10.5% | 7.7% | 6.5% | 56.6 | 231.4 | 33.3 | 199.4 |
|  |  |  |  |  |  |  |  |  |  |  |  |  |
|  | Canopy Temperature (°C) | | | | Canopy SPAD (SPAD Units) | | | | Average Greenness (Index Units) | | | |
| Treatment | Maris Piper | | Charlotte | | Maris Piper | | Charlotte | | Maris Piper | | Charlotte | |
|  | 5 L | 20 L | 5 L | 20 L | 5 L | 20 L | 5 L | 20 L | 5 L | 20 L | 5 L | 20 L |
| T_1/2_ | 19.5 | 18.1 | 19.1 | 17.9 | 43.8 | 44.6 | 47.1 | 46.8 | 0.30 | 0.32 | 0.27 | 0.40 |
| T_1_ | 18.3 | 18.0 | 18.1 | 17.7 | 42.3 | 44.8 | 48.4 | 46.9 | 0.33 | 0.34 | 0.27 | 0.34 |
| T_2_ | 18.0 | 17.7 | 17.7 | 17.3 | 42.0 | 44.2 | 48.2 | 46.2 | 0.31 | 0.36 | 0.27 | 0.33 |
|  |  |  |  |  |  |  |  |  |  |  |  |  |
|  | Average Hue (Index Units) | | | | Average NDVI (Index Units) | | | | Average PSRI (Index Units) | | | |
| Treatment | Maris Piper | | Charlotte | | Maris Piper | | Charlotte | | Maris Piper | | Charlotte | |
|  | 5 L | 20 L | 5 L | 20 L | 5 L | 20 L | 5 L | 20 L | 5 L | 20 L | 5 L | 20 L |
| T_1/2_ | 118.76 | 114.02 | 125.05 | 114.96 | 0.69 | 0.71 | 0.69 | 0.76 | 0.00 | 0.02 | -0.02 | 0.02 |
| T_1_ | 117.16 | 119.72 | 123.46 | 116.70 | 0.70 | 0.75 | 0.70 | 0.73 | 0.01 | 0.00 | -0.01 | 0.01 |
| T_2_ | 117.01 | 113.54 | 120.96 | 116.43 | 0.69 | 0.73 | 0.69 | 0.72 | 0.01 | 0.02 | 0.00 | 0.01 |
|  |  |  |  |  |  |  |  |  |  |  |  |  |
|  | Leaf Angle (°C) | | | | Light Penetration Depth (mm) | | | |  | |  | |
| Treatment | Maris Piper | | Charlotte | | Maris Piper | | Charlotte | |  | |  | |
|  | 5 L | 20 L | 5 L | 20 L | 5 L | 20 L | 5 L | 20 L |  |  |  |  |
| T_1/2_ | 40.20 | 41.40 | 41.47 | 43.69 | 197.89 | 290.45 | 121.49 | 381.20 |  |  |  |  |
| T_1_ | 42.32 | 41.62 | 41.70 | 44.37 | 191.61 | 247.47 | 122.32 | 281.59 |  |  |  |  |
| T_2_ | 41.91 | 41.08 | 42.66 | 41.28 | 264.54 | 346.65 | 162.76 | 331.63 |  |  |  |  |
